# Supplementary material for: Are carbon emissions trading and green financial instruments synergistic? -Comprehensive quantitative research based on content analysis
Source: PLoS One. 2024 Mar 7;19(3):e0298601. doi: 10.1371/journal.pone.0298601 (PMC10919601; doi:10.1371/journal.pone.0298601)
Supplement: S1 Data — (ZIP) [file pone.0298601.s001.zip › Data/地级市Z_F工作报告.zip!/░▓╗╒/╗┤▒▒╩╨/└┤╫╘╣1⁄2╓┌║┼í╛┬φ┐╦╩2╛▌═°í┐_2016.docx]

各位代表：
　　现在，我代表市人民政府，向大会报告政府工作，请予审议，并请市政协委员和其他列席人员提出意见。

****2015年及“十二五”：经济社会发展跃上新台阶****

　　2015年，全市人民在中共淮北市委的正确领导下，深入贯彻党的十八大和十八届三中、四中、五中全会精神，围绕加快城市转型、建设精致淮北主线，积极作为，真抓实干，统筹推进稳增长、促改革、调结构、惠民生，经济社会实现持续平稳发展。预计全年完成生产总值765亿元，增长4.5%；财政收入93.2亿元，增长2.1%，其中地方财政收入60.2亿元，增长14%；固定资产投资925亿元，增长10%；规模以上工业增加值490亿元，增长2%；城镇居民人均可支配收入25690元，增长8%，农村居民人均可支配收入10030元，增长10%；居民消费价格涨幅0.8%，城镇新增就业4.5万人，物价基本稳定，实现充分就业。
　　一年来，我们集中精力做了以下工作：
　　****（一）狠抓政策措施落实，经济实现稳定增长。****面对复杂多变的经济形势，采取一系列既利当前、更惠长远的重大举措，力促经济平稳有序运行。****持续增加有效投资。****以“九个一工程”为引领，突出抓好项目建设。453个重点项目完成投资417亿元，一批具有牵引作用的重大产业项目进展顺利。平山电厂一期工程1号机组并网发电，2号机组即将投产。海聚科技高速贴片生产线增至8条，投资25亿元的煤焦化综合利用二期投料试产。163个转型升级和技改项目加快推进，其中50个工业项目竣工投产。谋划重大项目187个，总投资1173亿元。争取国家专项建设基金11.98亿元。****大力促进社会消费。****加强流通主体培育，新增限额以上商贸流通企业82家。举办两届房地产展交会促销，全年销售商品房面积180万平方米，去库存60万平方米。开展“双百”“五进”活动，拉动消费20多亿元。全年实现社会消费品零售总额283.2亿元，增长12%。****积极扩大对外贸易。****着力培育外贸支柱企业，加大对90家重点外贸企业包保服务力度，全市外贸进出口总额5.5亿美元，较上年略有增长。设立市口岸办，与上海铁路局签订建设青龙山铁路枢纽“无水港”合作协议。****全力以赴帮扶企业。****安排财政资金1.5亿元，实施稳岗补贴政策，惠及职工18.2万人。失业保险费率由3%降至2%，减轻企业缴费负担1.12亿元。全面落实结构性减税政策和涉企收费清单制度，减免企业税费10.7亿元。深化“干部帮联企业、打造精良产业”行动，一企一策帮助企业解决实际问题。通过开展百名金融顾问服务百家企业活动，推进“4321”政银担风险分担机制，设立1.6亿元续贷过桥资金等举措，有效化解了中小企业融资难融资贵问题。深化与淮北矿业、皖北煤电、淮海实业、中煤矿建等省属企业合作发展。实施企业培育工程，规模以上工业企业增至799家。
　　****（二）加大结构调整力度，转型升级取得新成效。****制定调转促“496”实施方案，开展9大重点工程专项行动，全面打响调转促攻坚战。****工业结构不断优化。****非煤产业增加值占全市工业的70.8%，同比提高5.9个百分点，对财税贡献首次超过煤炭行业。设立1亿元的产业投资引导基金，继续加大对“双新”产业扶持力度。高新技术产业和战略性新兴产业分别实现产值465.5亿元和360亿元，为加快调转促奠定了良好基础。规模以上民营工业企业增加值占全市工业的比重达66.9%，同比提高6.1个百分点。新增5件中国驰名商标，相当于建市以来的总和。****服务业发展提质增效。****服务业完成增加值238亿元，占全市生产总值的31%，较上年提高2个百分点，服务业对经济增长的贡献达到53.5%。中易物流园、隋唐运河古镇文化旅游等项目加快建设，口子文化博览园主体工程完工。4个电商产业园区建成运营。成功举办第四届食博会、第六届石榴文化旅游节和第二届葡萄采摘节。全年旅游综合收入38.9亿元，增长15.2%。****现代农业发展步伐加快。****小麦单产保持超千斤水平，粮食生产“十二连丰”。新增设施蔬菜种植面积1.02万亩、水果种植面积8500亩。新增土地流转面积3万亩，家庭农场增至1169家，农民合作社发展到1206家。培育市级以上农业产业化龙头企业141家，农产品加工业产值542亿元，增长6.4%。凤凰山、榴园、高岳获批省级现代农业示范园区。
　　****（三）深化改革扩大开放，发展活力不断释放。****坚持用改革的办法应对困难挑战，以开放拓展发展空间。****重点领域改革实现突破。****获批全省唯一的国家中小城市综合改革试点市。市政府职能转变和机构改革工作顺利完成。加大简政放权力度，市级权力事项精简49.1%，市、县（区）、镇（街道）三级政府权责清单体系建立并公布实施。基层医药卫生体制综合改革持续深化，市级公立医院综合改革试点全面启动。颁发农村土地承包经营权证2.1万份。按期完成机关事业单位工资调整和市级公务用车制度改革。在县以下机关建立公务员职务与职级并行制度，启动机关事业单位养老保险制度改革。建立以政府债券为主体的举债融资机制，将政府债务纳入全口径预算管理。完善市属国有企业分类考核评价办法，推进国有资产集中统一监管。投入资金1.4亿元，实施政府购买服务项目60个。集中新建政务服务、公共资源交易、房产交易与不动产登记、金融服务、信访接待五个便民利企服务中心，“一站式”服务水平进一步提升。****金融创新成效显著。****市建投集团顺利改革转型，全年融资突破50亿元，首次通过中国银行间协会和上海证交所各发债20亿元，首次从东亚银行获得6亿元外资贷款。全市直接融资106.6亿元，实现历史性突破。口子酒业在上交所主板上市，3家企业成功登陆“新三板”，26家企业在省区域四板市场挂牌。****招商引资强力推进。****坚持市级领导带头招商，强化精准招商、以商招商和产业链招商，引进并开工规模以上项目103个，其中投资3亿元以上项目10个。中国供销淮北农产品批发市场项目签订建设协议，年产18万吨煤焦油加氢及5万吨针状焦联产和相山海螺日产4500吨新型干法水泥熟料生产线项目成功引进，投资20亿元的金龙光电触摸模组、投资5.1亿元的传化公路港物流等一批重大项目全面开工，上海电气、浦发集团投资的生物质发电项目加快建设，20万吨混合芳烃精制及苯基合成材料一期项目即将建成，相邦铝基复合材料一期建成投产，纳税超过千万元。****创新创业持续拓展。****推进企业主体、创新载体和产学研一体建设，加快科技成果转化，增强创新驱动力。新增国家高新技术企业19家，发明专利授权量增长107.4%，获批国家知识产权优势企业5家。成功创建凤凰山国家农业科技园区、烈山区国家可持续发展实验区，濉溪经济开发区获批省铝基新材料高新技术产业基地。出台“1+6”推进大众创业政策，推动创业带动就业。实施创业富民提升工程，开展创业培训3610人，提供创业指导3741人次，培育初创企业216户，带动就业1.5万人。淮北技师学院获批筹建。落实“三证合一、一照一码”等改革措施，新登记注册私营企业3908户、增长21.3%，新增个体工商户12431户、增长19.5%。****智慧城市一期工程进展顺利。****社会服务管理信息化“一站通”平台在全市174个社区实现全覆盖，延伸到14个村，169项与群众密切联系的事项实现网上办理。市直部门权力清单涉权事项4942项、县区涉权事项8147项即将全部上线运行。建立大数据库，入库各类数据3.68亿条。设立智慧城市产业投资基金，与北大方正合作推进淮北方正智谷科技园建设。智慧城管和平安工程二期加快推进。
　　****（四）统筹推进城乡建设，精美城市形象进一步彰显。****以文明城市创建为抓手，完善基础设施，强化城市管理，改善农村人居环境。****重大基础设施建设持续推进。****淮北至萧县北客车联络线和符夹线扩能关键节点工程达到序时进度，青阜线电气化改造启动。完成城乡公路交通建设投资13亿元，濉唐路一期工程完成，与泗许高速互联互通，打开了城区南大门；合相路一期主体工程完工，拓展了城区向西延伸的空间，解决了多年梦想解决的省道202穿越濉溪县城的问题。萧淮路中段建成通车，市区至煤化工基地快速通道、孟山南路连接线分别完成工程总量的69%和61%。淮水北调及市级配水工程全面开工建设，已基本具备通水条件。220千伏飞来峰及110千伏溪河、西园、皖苏输变电工程竣工投运。****城市建设步伐加快。****市区两级投入资金2亿元，改造提升老城区配套设施。东山北路改扩建工程、西山南路建成通车。加快推进13条断头路建设，鹰山南路、光明路顺利贯通。古城路等4条道路人行道改造，迎宾大道环岛、淮海路公交候车亭改建，孟山北路等5条道路路灯提升工程全面完成。升级改造纺织苑、南黎农贸市场。新建、改造供水与燃气管网106公里，完成地下管网普查2600公里，年新增中石油天然气供应2000万立方米。实施城市增绿提升工程，绿化城市道路12条，新建公园游园和街头绿地16个，新增城镇绿地193万平方米，建成城市绿道57公里。南黎公园、仁和公园、桓谭廉洁文化公园、东湖花海、长山南路绿化成为新的城市景观。引导主城区优质公共资源向东部新城区转移，市职教园区二期工程进展顺利，市一中东校区、市人民医院东院区、“创客驿站”大学生服务中心完成规划设计，即将开工建设。****城市管理不断加强。****“多城联创”取得积极进展，顺利通过全国绿化模范城市国家验收和全国无障碍建设先进城市省级复核。开展市容、交通秩序、户外广告等专项治理，市区主次干道全部实现市场化清扫保洁，餐厨垃圾集中处置项目加快建设，划定相山区露天烧烤禁烧区，城市环境更加整洁美观。加强主城区公共停车资源综合管理，新划定临时公共停车场13处，新增各类临时停车位1500多个，缓解了道路拥堵和停车难。新投放公共自行车3100辆，站点布局覆盖市区和濉溪县城，市民出行更加便捷。全省物业管理工作现场会在我市召开。****美丽乡村建设有序实施。****加快第二批11个中心村建设，实施村庄道路、绿化美化亮化等项目151个，竣工136个。第三批11个中心村规划建设全面启动。榴园村入选全国特色景观旅游名村和全省绿色村庄示范村，“四季榴园”创建国家4A级景区顺利通过评审。蒙村、郭王村跻身全省美丽宜居村庄。
　　****（五）突出抓好污染防治，生态环境持续改善。****成立市环保委，实行治超、治污、治安、治税“四治”联动，统筹推进环境污染治理。****大气、水污染防治取得新成效。****集中整治交通运输扬尘、机动车污染、燃煤锅炉、城市建设扬尘、石料厂物料堆场搅拌站、餐饮油烟等领域污染。超额完成省定“黄标车”淘汰任务，秸秆禁烧工作取得明显成效，全市PM10平均浓度降至90微克/立方米，同比下降12微克/立方米，顺利完成省控目标。开展城市黑臭水体治理摸底调查，相阳沟、跃进河等城区沟河清淤工程基本完成。城市集中式饮用水源地水质达标率100%。新建污水处理厂2座，新建污水管网42公里，我市污水处理设施建设运行考核居全省第三。****生态建设加快推进。****继续实施森林增长工程，完成造林绿化3.1万亩，建成森林长廊43.6公里，森林覆盖率达到20.7%。投资1.09亿元，完善南湖核心景区配套设施，生态环境和景观效果进一步提升。完成东湖景区一期绿化1800亩，启动中湖治理，谋划申报河湖连通与水生态环境综合整治工程。
　　****（六）大力改善民计民生，社会治理效能和服务群众水平有效提升。****全市用于民生支出108.7亿元，占财政总支出的83%，34项民生工程全面完成，城乡居民得到了更多实惠。****就业和社会保障工作持续加强。****实施积极就业政策，大力扶持高校毕业生和困难群体就业，城镇登记失业率控制在4.1%以内。企业离退休人员养老金人均月增184元，城乡居民基础养老金标准提至70元。发放家庭经济困难学生补助金2580万元。在全省率先开展重特大疾病医疗救助试点，推进省际异地就医联网结算。开展大病保险试点，报销比例提高10至15个百分点。新开工各类保障性住房和棚户区改造安置住房11136套，基本建成12765套。在全省率先出台一系列改善市民居住条件的优惠政策，棚改货币化安置顺利实施。1435户农村危房改造全部竣工。****社会事业协调发展。****继续实施第二期学前教育三年行动计划，新建改扩建幼儿园7所。落实进城务工人员随迁子女就读义务教育阶段学校入学政策，推进义务教育资源合理配置，引导教育资源向农村和薄弱学校倾斜。出台创新发展现代职业教育实施意见，深化职业教育市级统筹改革。公共卫生保障能力不断增强，基层医疗卫生机构条件和服务水平明显提升。新农合工作综合考评居全省第一。市歌《淮北是我家》广为传唱。我市健儿在全国首届青运会上获得一金一铜。人口计生工作全面完成省下达目标任务。妇女儿童、青少年、老龄等事业得到加强，民族宗教、外事侨务、对台、档案、地方志、气象、防空防震等工作取得新进展。****社会大局和谐稳定。****成功举办全国农村社区建设试点工作推进会。“六五”普法圆满收官。扎实推进平安淮北建设，社会治安整体防控能力显著增强，基层综治维稳信访工作中心（站）全面建成。建立涉法涉诉信访依法终结制度，推进网上信访，完善信访和调解联动工作体系。由市级领导牵头包案，加大对房地产领域信访突出问题的处理力度。健全农民工工资专户制度，有效解决建设领域拖欠农民工工资问题。严格落实安全生产责任制，各类事故起数和死亡人数连续3年实现双下降。加大食品药品安全治理整顿力度，保障群众饮食用药安全。
　　****（七）着力转作风提效能，政府自身建设不断加强。****围绕“三严三实”专题教育，深入开展精细作风建设。严格执行中央八项规定，规范公务接待，全市“三公”经费支出下降12.3%。坚持依法行政，在全省率先建立市政府重大事项决策制度体系。自觉接受人大法律监督、政协民主监督和社会舆论监督，办理人大代表建议113件、政协委员提案185件，满意或基本满意率达到98%以上。不断深化政务公开、网络问政，办理市长信箱和网上留言1088条，办结率98%以上，荣获全国网民留言办理工作先进单位称号。加强行政执法和效能监察，加大审计监督力度，严肃查处违法违纪违规行为。
　　各位代表！回首过去五年，我们主动适应经济发展新常态，积极应对各种困难挑战，凝心聚力，锐意进取，开创了转型发展和精致淮北建设新局面。
　　****这五年，综合实力大幅提升。****全市生产总值连跨三个百亿元台阶，年均增长9.7%。财政收入年均增长5.9%，规模以上工业增加值年均增长11.2%，固定资产投资3493.7亿元，是上一个五年的3.1倍。濉溪县进入中国县域经济最具发展潜力百强县（市）榜单。相山区获评全国食品工业强区。
　　****这五年，产业结构逐步优化。****三次产业结构比例由8.8:64.6:26.6调整为8:61:31。非煤产业成为拉动全市经济增长的重要引擎，战略性新兴产业加速培育。连续两次获得全国科技进步先进市荣誉称号，国家高新技术企业较“十一五”末增长了6倍。顺利通过国家循环经济试点示范城市验收。
　　****这五年，发展基础不断夯实。****累计完成重点项目投资2018亿元，安徽矿机装备、大唐虎山电厂、华孚色纺工业园一期、思朗食品等一批重大产业项目相继建成投产。安徽（淮北）新型煤化工合成材料基地建设上升为省级战略。淮北至萧县北客车联络线加快推进，完成火车站改扩建工程，泗许高速和济祁高速淮北段建成通车，南坪港实现通航，综合交通体系日益完善。
　　****这五年，发展活力持续迸发。****国家中小城市综合改革试点和新型城镇化试点工作全面展开，户籍制度改革走在全省前列。金融服务体系不断完善，交行、招行、马鞍山农商行入驻，成立首家村镇银行，组建淮矿集团财务公司，在全省率先实施农村土地承包经营权抵押贷款试点。民营经济加快发展，占全市生产总值比重五年提高10.9个百分点。对外开放迈向新台阶，累计引进亿元以上项目343个，利用省外资金1332亿元。实际利用外资22.8亿美元，年均增长25.7%。
　　****这五年，城乡面貌深刻变化。****户籍人口城镇化率达到61%，年均提高1.3个百分点。入选国家智慧城市、餐厨废弃物综合利用和无害化处理试点城市。再次跻身全国文明城市提名资格城市行列。新增森林面积21.35万亩。“三线三边”治理成效显著，打造一批“村容整洁、生态宜居、特色鲜明、生产发展”的美丽乡村。
　　****这五年，人民生活显著改善。****城乡居民收入年均分别增长11.1%和13.7%。民生工程考核连续多年位居全省第一方阵。获评全国创业先进城市。城镇新增就业25.5万人，农村劳动力转移就业45.2万人。连续多年被评为全省社会治安综合治理工作先进市，人民群众安全感和幸福感进一步提升。
　　各位代表！看似寻常最奇崛，成如容易却艰辛。在经济下行压力不断加大、煤炭价格断崖式下跌的情况下，2015年及“十二五”取得了来之不易的发展成就，这是省委、省政府和市委正确领导的结果，是市人大、市政协与社会各界大力支持的结果，是全市人民创业创新、团结奋斗的结果。在此，我代表市人民政府，向全市人民，向人大代表、政协委员，向各民主党派、工商联、无党派人士和人民团体，向各位老领导、老同志，向驻淮解放军、武警官兵和政法干警，向关心和帮助淮北发展的社会各界人士表示诚挚的感谢，并致以崇高的敬意！
　　在总结成绩的同时，我们也深切感到，前进的道路上还存在不少困难和挑战。经济总量不大、结构不优，煤炭企业生产经营困难加大，战略性新兴产业和现代服务业发展不足，财政收支矛盾突出。城市棚户区改造任务十分繁重，房地产领域历史遗留问题需要持续化解，农村贫困人口脱贫尚需加大帮扶力度。政府工作中也存在差距和不足，少数公职人员思想观念、工作能力不能适应新常态，工作中还存在不会为、不敢为等问题。对此，我们将采取积极措施，认真加以解决。

****“十三五”：努力实现全面建成小康社会奋斗目标****

　　“十三五”是全面建成小康社会的决胜阶段，也是推动我市发展转轨、产业转型、城市转向、动力转换的关键时期。当前，国际金融危机深刻影响还在继续，世界经济仍处在深度调整期，国内经济下行压力加大，可能会经历一个L型增长阶段，我市依靠煤炭支柱产业支撑较高发展速度不再可能，新的增长动力还不足弥补传统增长动力弱化造成的缺口。同时也要看到，未来五年，全球新一轮科技革命和产业变革正在创造历史性机遇，创新引领发展潮流涌动。特别是随着国家“一带一路”、京津冀协同发展、长江经济带、中原经济区和我省淮河流域综合治理与振兴发展等重大战略的深入实施，以及我市高铁项目即将建成，淮北处在京沪之间、四省交界、全国居中的区位优势更加凸显，要素聚集能力和开放发展水平将进一步提升。我们既要强化忧患意识，树立底线思维，又要强化机遇意识，坚定发展信心，保持战略定力，勇于破解发展难题，厚植发展优势，继续为淮北转型发展奠定坚实基础。
　　根据市委七届十一次全会通过的《关于制定国民经济和社会发展第十三个五年规划的建议》，****我市“十三五”发展的指导思想是：****高举中国特色社会主义伟大旗帜，全面贯彻党的十八大和十八届三中、四中、五中全会精神，以马克思列宁主义、毛泽东思想、邓小平理论、“三个代表”重要思想、科学发展观为指导，深入贯彻习近平总书记系列重要讲话精神，按照“五位一体”的总体布局和“四个全面”的战略布局，牢固树立创新、协调、绿色、开放、共享的发展理念，着力提高发展的质量和效益，以城市转型为主线，深入推进国家中小城市综合改革试点，探索建立引领经济发展新常态的体制机制和发展方式，建设精致淮北，全面建成小康社会。
　　今后五年经济社会发展的主要目标是：
　　——****经济持续健康较快增长。****在提高发展平衡性、包容性、可持续性的基础上，保持经济中高速增长，地区生产总值年均增速超过全省平均水平。到2020年，人均地区生产总值突破1万美元。
　　——****产业升级取得实效。****到2020年，三次产业结构进一步优化，以战略性新兴产业为先导、先进制造业为主体、现代服务业大发展的现代产业格局基本形成，产业迈向中高端水平。
　　——****创新创业活力增强。****创新创业体系更趋完善，形成适应创新驱动发展的制度环境。民营经济发展与大众创业、万众创新取得新突破。园区科技发展平台更加坚实，企业创新主体地位更加突出，科技进步对经济增长的支撑作用持续增强。
　　——****改革开放不断深化。****国家中小城市综合改革试点取得积极进展，重点领域和关键环节改革取得明显成效，资源型城市转型的体制机制不断完善，部分改革试点走在全省乃至全国前列。区域合作、招商引资与对外贸易的质量水平不断提高。
　　——****城乡建设更加精美。****新型城镇化试点深入实施，美丽乡村建设扎实推进，城乡空间布局进一步优化，城市对人口的集聚能力不断增强，山水生态宜居城市特色更加彰显。
　　——****生态环境日趋优良。****资源能源集约节约利用，能源和水资源消耗、建设用地、碳排放总量和强度得到有效控制，主要污染物达标排放，空气质量持续改善，生产生活方式绿色水平不断提升。园林城市和绿化模范城市建设走在全省前列。
　　——****社会民生持续改善。****城镇居民收入与经济增长同步，农村居民收入快于经济增长。贫困人口提前实现整体脱贫。覆盖城乡居民的公共服务体系更加健全。市民整体素质明显提高，实现全国文明城市争创目标。法治淮北建设取得显著进展，社会治理水平不断提高，社会更加和谐稳定。
　　重点把握五个方面：
　　****（一）坚持创新发展，培育转型升级新动能****
　　实施战略性新兴产业集聚发展工程，到2020年，全市1-2个产业基地进入全省战略性新兴产业集聚发展基地。实施传统产业改造提升工程，推进先进技术和智能化装备应用，促进煤炭、电力、装备制造、纺织服装、新型建材五大传统产业向价值链高端发展。实施服务业加快发展工程，推进生产性服务业向专业化和价值链高端延伸，促进生活性服务业向精细化和高品质发展。实施农业现代化推进工程，构建现代农业经营体系、生产体系和产业体系，走三次产业融合发展之路。实施质量品牌升级工程，以品牌质量促进产业升级。实施创新驱动发展工程，创建国家创新型城市。明确各开发园区主导产业和发展方向，打造特色产业集群，加快产城融合。推动大众创业、万众创新，鼓励发展众创、众包、众扶、众筹。实施“互联网+淮北”行动计划，推进互联网与经济社会深度融合。实施人才高地建设工程，建立以企业为主体、产业为纽带、政府加强引导的人才培育机制。实施民营经济提升工程，鼓励民营企业依法进入更多领域，依法保护企业家财产权和创新收益。
　　****（二）坚持协调发展，形成统筹推进新格局****
　　实施“416”城市空间发展战略，形成“一主一次，南北联动”的城市发展格局。深化美丽乡村建设，全市镇级政府所在地和自然村环境整治实现全覆盖。实施县区经济振兴工程，到2020年，县区生产总值、财政总收入分别占全市的80%和55%以上。推进交通、水利等重大基础设施和智慧城市建设。发展城乡公共交通。坚持城乡同治、全域共建，统筹推进城乡文明一体化发展。培育精深文化，提升城市文化软实力。推动军民融合深度发展，实施一批军民融合产业项目。
　　****（三）坚持绿色发展，塑造生态文明新优势****
　　合理布局城市生产、生活、生态空间。发展生态循环农业，推行企业循环式生产、产业循环式组合、园区循环式改造，争创国家循环经济示范城市。坚决打好大气、水、土壤污染防治攻坚战，争创全国环境保护模范城市。推进农村生活污水、垃圾处理，加快河湖、塌陷区综合治理。促进资源集约节约高效利用。
　　****（四）坚持开放发展，拓展对内对外合作新空间****
　　全面加强区域合作，主动对接“一带一路”、长江经济带、中原经济区建设。瞄准长三角、珠三角和台湾等重点区域，实施精准招商。加强与央企和知名民企合作，积极承接北京非首都功能疏解转移和上海产业转移。扩大外贸规模，优化外贸结构，支持企业加快走出去，积极参与国际产能和装备制造合作。
　　****（五）坚持共享发展，建设和谐幸福新淮北****
　　发展学前教育，促进城乡义务教育均衡发展，普及高中教育，大力发展职业教育，推进高等教育发展。坚持以创业带动就业，持续增加城乡居民收入。健全城乡社会保障体系，基本实现法定人员全覆盖。完善突发公共卫生事件应急体系。实施“医养结合”等新型养老模式，鼓励社会力量兴办健康和养老服务业。创新计划生育管理，提高人口发展协调性。实施“全民健身、健康淮北”行动。深入推进扶贫开发，到2017年，确保完成全市脱贫任务。

****开局之年：奋力谱写转型发展和精致淮北建设新篇章****

　　今年是全面建成小康社会决胜阶段的开局之年，也是推进结构性改革、全面实施调转促“496”实施方案的攻坚之年。我们要适应、把握和引领经济发展新常态，坚持稳中求进工作总基调，坚持扩大开放，招商引资，增加投资，在继续扩大有效需求的同时，着力加强供给侧结构性改革，去产能、去库存、去杠杆、降成本、补短板，增强持续增长动力，着力提高经济发展质量和效益，着力保障改善民生，努力实现“十三五”发展良好开局。****经济社会发展的主要预期目标是：****全市生产总值增长7%左右，财政收入增长2%，固定资产投资增长10%以上，规模以上工业增加值增长7%，社会消费品零售总额增长12%，外贸进出口总额增长5%，城乡居民人均可支配收入分别增长8%和10%，城镇新增就业4万人，城镇登记失业率控制在4.2%以内，节能减排完成年度目标任务。
　　重点做好十个方面工作：
　　****（一）加快调结构转方式促升级，力促经济在稳定增长中提质提效****
　　****千方百计支持实体经济发展。****强化经济运行调节和监测预警，对企业分类指导。积极开展企业降本增效专项行动，重点在降低制度性交易成本、企业税费、社会保险费、财务成本、电力价格、物流成本等方面，打出“组合拳”，帮助企业应对当前困难，保持竞争优势。积极稳妥化解过剩产能，在优化存量中提升产能利用率和市场竞争力。支持在淮省属企业深化改革，创新发展第三产业和新业态，实现转型升级。继续深化“干部帮联企业，打造精良产业”行动，搭建银企对接、产销对接平台，帮助开拓市场，稳定企业发展。
　　****加快推动工业转型升级和开发园区建设。****围绕创新驱动、项目拉动、龙头带动、园区载动，推进新型煤化工合成材料、铝基新材料、电子信息、绿色食品、高端装备制造、生物医药等战略性新兴产业集聚发展。加快推进相邦铝基复合材料二期、金龙光电触摸模组、淮北北大方正智谷科技园等项目建设，力促战略性新兴产业产值增长15%。推动七大开发园区抓产业规划，抓龙头企业，抓设施配套，抓科技创新，抓争创省级基地，力争成为工业增长和产业转型升级的新引擎。开展制造业升级专项行动，推动新一轮技术改造，在装备制造、食品等领域实施“互联网+”创新示范试点工程，促进传统产业向价值链高端发展。组织实施年度转型升级和技术改造项目160个，完成工业技改投资190亿元，全面提高产品技术、工艺装备、能效环保水平。加快培育一批安徽名牌和中国驰名商标。促进中小企业“专精特新”发展。继续开展“抓在建、促投产”活动，推动企业“小升规”，新增规模以上工业企业80户。
　　****优先发展服务业。****加快现代物流、信息咨询、科技服务等生产性服务业发展，推进文化旅游、商贸流通、家政服务、体育休闲、健康养老等生活性服务业发展，创新发展电子商务等新型商业业态。顺应消费结构加快升级和个性化定制消费的趋势，积极培育新兴消费，扩大传统消费，发展新的消费模式。争取列入省首批信息消费试验区。继续实施促进居民住房消费的优惠政策，鼓励农民进城购房，鼓励市民改善住房条件，鼓励发展个人投资住房租赁业，鼓励房地产开发企业适当降低商品住房价格，积极消化房地产库存，促进房地产稳定发展。加快传化公路港物流、青龙山铁路无水港、邮政快递物流园等项目建设。新增限额以上商贸流通企业50家，服务业增加值占全市生产总值比重达到32.5%以上。
　　****强化科技创新支撑。****完善建设创新型城市总体方案，围绕调转促，实施创新主体培育、“双创”平台打造、产学研开放创新、产业技术升级和科技金融结合六大行动计划。加强与市内外高等院校和科研院所的合作。争创国家高新技术产业基地1家，新增创新型（试点）企业3家，实现高新技术产业产值480亿元，高新技术企业占规模以上工业企业的比重达到10.5%，万人有效发明专利拥有量达到3件。
　　****（二）坚定不移推进招商引资和项目建设，积极培育新的经济增长点****
　　****大力开展招商引资。****积极与央企对接，及时把握央企投资和产业布局动态，力争引进一批体量大、质量高、牵动性强的合作项目。坚持与知名民企、台企和外企深化合作，瞄准长三角、珠三角、京津冀、台湾地区等产业转移重点区域，围绕现代服务业和高端装备制造、电子信息、绿色食品等新兴产业，制订有针对性的招商方案，组织举办专题招商推介，开展“一对一”上门招商、“点对点”对接项目。密切跟踪北京、上海等地产业转移新动向，依托各种人脉关系、客商资源，力争尽快掌握京沪两地产业转移的范围、对象和批次，结合我市需求，筛选确定承接的重点产业、重点企业、重点项目。利用新型煤化工产业发展研讨会、铝基新材料产业发展研讨会、食博会、石榴文化旅游节和葡萄采摘节等节会平台，加大产业招商力度，加快引进一批战略投资者。调整招商引资考核办法，强化县区、部门、开发区招商引资主体责任，完善重大产业项目调度和协调推进机制，力促万达商业广场、中国供销淮北农产品批发市场等一批项目尽快落地建设。确保全年利用市外资金增长10%以上，力争引进10个投资规模超5亿元的大项目。
　　****强化项目支撑带动。****今年计划安排重点项目506个，完成年度投资401亿元。全面实施“四督四保”制度，完善协调推进机制，突出抓好以“九个一工程”为重点的项目建设，以项目带动产业发展。开工建设单机135万千瓦机组的平山电厂二期国家示范工程、18万吨煤焦油加氢及5万吨针状焦联产项目，加快推进口子东山产业园、华孚色纺50万锭工业园二期、相山海螺日产4500吨新型干法水泥熟料生产线等项目建设，确保煤焦化综合利用二期、年产20万吨混合芳烃精制及苯基合成材料一期项目全面投产，华中天力、浩丰实业等达产达效。
　　****加紧谋划申报项目。****紧抓我省实施淮河流域综合治理与振兴发展的战略机遇，着力在城乡建设、交通水利、产业发展、采煤沉陷区治理与水环境综合整治等方面谋划实施一批项目，争取列入省支持范围。积极对接国家重大工程包谋划项目，继续跑省跑部申报国家专项建设基金，新增入库储备重大项目200个以上，总投资超过1000亿元。
　　****加快发展民营经济。****加大对大众创业的财税支持和金融服务，健全完善创业辅导、人才培训、信息咨询等公共服务体系，激发全民创业热情，确保新增私营企业2150户、个体工商户4800户以上，民营经济增加值占全市生产总值比重同比提高一个百分点。扶持百家骨干民营企业做大做强，支持有实力的民营企业兼并重组，鼓励企业以增资扩股、股权转让等形式引进战略投资者，推进各类资本交叉持股或合作投资。以民办公助等形式，支持创新工场等新型孵化器建设。
　　****强化要素保障。****认真研究国家产业政策和投资重点，坚持以项目争取资金。积极稳妥推进PPP等市场化投融资模式，多渠道筹措建设资金。积极争取用地指标，实施整体推进农村土地整治，加大闲置低效用地清理和再利用力度，推进工矿废弃地治理，保障重大项目用地需求。推动招才引智和本土人才开发并举，创造吸引人才、留住人才和有利于人才创新创业的宽松环境。
　　****（三）推进重点领域改革攻坚，构建有利于转型发展的体制机制****
　　****推进国家中小城市综合改革试点。****围绕增强城市资源聚集能力、人口吸纳能力和综合承载能力，突出“产、钱、地、人、治”，全面推进产城融合、城市投融资、土地要素、民生保障、城市治理等重点领域改革，着力解决制约城市转型发展的深层次矛盾。
　　****深化行政体制改革。****持续推进“放管服”结合，深化权力清单和责任清单制度建设，制定政府权力运行监督管理细则，实施清单动态管理。完善市、县（区）、镇（街道）三级政府权力清单平台建设，实现所有权力事项网上公开运行全覆盖。
　　****改革城市规划建设管理体制。****稳步推进城市规划建设管理重心下移，分级负责，把主体责任落实到县区，基础工作分解到镇、街道，建立条块结合、权责利统一的长效机制，调动县区抓城市工作的积极性和创造性。
　　****持续推进财税金融改革。****进一步完善预算体系，加大政府性基金、国有资本经营预算与一般公共财政预算的统筹力度。推进全口径预算编制，将政府所有收支纳入预算管理。规范政府性债务管理。稳步推进税收征管体制改革。组建省股权托管交易中心皖北融资中心。创新开展“信贷+”融资服务。加大企业上市（挂牌）工作推进力度，鼓励优质企业利用股权和债权拓宽融资渠道。
　　****创新国有资产管理体制。****深化国企改革，加强资金资产资源管理，实现由管资产向管资本转变。完善国有资本经营预算制度，健全预算调入机制，规范收益收取，确保国有资产保值增值。
　　****（四）积极推进新型城镇化，促进全域统筹发展****
　　****加强重大基础设施建设。****积极推进淮北-阜阳、淮北-合肥城际铁路前期工作。加快推进符夹线扩能、青阜线电气化改造工程，力争高铁联络线主体工程年底前完工。开工建设合相路、古毛路、淮六路与濉唐路4条道路二期工程。确保6月底前，市区至煤化工基地快速通道、萧淮路淮北段路面改善工程建成通车。实施浍河南坪至临涣闸段航道整治。南坪船闸主体工程完工。确保110千伏光明变、口子变输变电工程投运。
　　****提升城市规划建设管理水平。****将新修编的城市总体规划上报国务院审批，完善各类修建性详规和专项规划，促进市、县“多规合一”。以东部新城建设为重点，优化城市功能布局与空间结构，不断彰显“一带双城三青山、六湖九河十八湾”的精美城市风貌。高标准规划建设采煤沉陷村庄搬迁集中安置片区，与百善镇规划建设相统一，打造宜居宜业的城市南部次中心。加快地下综合管廊等规划编制，积极争取进入国家第二批海绵城市建设试点。落实城市控制性详规向市人大备案制度。实施老城区主次干道、背街小巷、地下管网等设施改造升级，新建、改建一批城区菜市场。抓好龙山南路等续建工程建设，加快实施泉山北路等10个符夹铁路道口改造工程，打通方安路等老城区12条断头路，建成淮海路人行天桥，畅通城区交通微循环。完善东部新城路网、水网、电网、排污治污等基础设施和住宿餐饮、休闲娱乐等商业服务设施配套，提升新城区服务功能。拓宽改造梧桐中路，规划建设东部新城与老城区间的快速通道。抓好市人民医院东院区、一中东校区、职教园区二期工程建设，建成“创客驿站”大学生服务中心。开通濉溪至市职教园区109路新公交班线。开工建设城市规划馆和会展中心。实施太山路等14条新建道路绿化及东湖景区二期绿化，推进相阳沟、老濉河南段、萧濉新河北段等河渠绿化，完成城镇绿化190万平方米。新建儿童公园、花山公园。深入推进文明城市创建，加强城市环境综合整治，进一步提升城市形象和管理水平。建设智慧城市二期工程，应用大数据促进城市管理科学化、精细化、便民化。推动城乡环卫一体化全覆盖。加强生活垃圾分类回收处理，启动餐厨废弃物收运体系建设。全面推进物业管理规范化、现代化。
　　****加快推进农村人口市民化。****深入实施城乡户籍一元化管理改革，全面放开市区、县城和建制镇落户限制，完善居住证公共服务功能。探索建立城乡基本公共服务衔接并轨机制，推进“五有”并轨。落实进城农民土地承包经营权、宅基地使用权和集体资产收益分配权，注重开发适合农业转移人口的就业岗位，提高农民进城落户积极性。
　　****加快建设美丽乡村。****全面完成第三批11个中心村项目建设，启动第四批中心村规划建设。持续加强 “三线三边”环境综合整治，推进“三治三增三提升”向镇、村延伸，全面改善农村生产生活条件。结合精准扶贫，大力实施“一村一品”兴业富民工程，扶持农村特色产业发展。
　　****（五）大力推进农业现代化，夯实“三农”发展基础****
　　****深化农村综合改革。****推进土地适度规模经营，基本完成农村土地承包经营权确权登记颁证工作。稳步推进农村土地承包经营权贷款试点，探索开展农民住房财产权抵押贷款、农村集体资产股份制、农村公共服务运行维护等改革试点。深化供销社改革。
　　****提升发展现代农业。****加快农业水利建设，实施农业机械化示范创建工程。加强农业科技创新，推广农业物联网技术。开展粮食绿色增产模式攻关，建立135万亩核心示范区，扩大设施蔬菜种植面积，发展优质水果种植，推进标准化规模养殖。实施绿色增效工程，推进作物秸秆综合利用和农业标准化生产。加快凤凰山国家级农业科技园区和4个省级现代农业示范区建设。
　　****扶持发展新型农业经营主体。****采取财政扶持、信贷支持等政策措施，加快培育骨干龙头企业+合作社+家庭农场的新型农业产业化联合体。规范农民合作社发展，提高家庭农场生产经营能力。大力发展主体多元、形式多样、竞争充分的农业社会化服务体系。发展农产品电子商务，在知名电商平台筹建淮北农产品特色馆。
　　****（六）持续推进精深文化建设，以文化内涵提升城市品位****
　　****深入实施文化惠民工程。****整合公共文化资源，完善公共文化服务体系，加强乡镇综合文化服务中心和中心村农民文化乐园建设。构建覆盖城乡的文化体育基础设施网络，推动城乡基本公共文化服务标准化、均等化发展。加强广播电视村村通工程管护，探索完善农村公共文化设施 “建管用”机制。继续开展市民文化节和文化惠民消费季等文化活动。
　　****发展壮大文化产业。****深化文化体制改革，推进文化与旅游、教育、科技、体育等行业的快速融合，增强文化产业发展活力。提升发展新闻出版、广告演艺等传统文化产业，积极发展创意设计、网络传媒等新兴文化产业。加快隋唐运河古镇文化旅游、国购汽车文化园等项目建设，推动南湖等旅游景区创建国家4A级景区，口子文化博览园建成开放。加快传统媒体与新兴媒体融合发展，扩大和引领文化消费。
　　****传承创新特色文化。****深入挖掘红色文化、运河文化、好人文化、石榴文化资源，打造一批具有淮北特色、知名度高的文化品牌。编制红色旅游发展规划，开通红色旅游线路。放大运河世遗效应，加大柳孜运河遗址保护和旅游开发力度。
　　****（七）持续推进绿色低碳循环发展，建设山清水秀天蓝的美好家园****
　　****加强环境保护和生态建设。****坚持“四治”联动推进环境整治，持续加大治霾治污力度，落实主要污染源监管措施，全部关闭燃煤小锅炉，提标改造火电、水泥等高污染行业企业，标本兼治推进秸秆禁烧，确保PM10平均浓度继续下降。加强污水处理设施改造升级，推进老濉河、浍河等主要流域及龙支河水环境综合治理。强化畜禽养殖污染和农业面源污染治理，加强饮用水源地保护。实施森林增长工程和石质山场绿化景观提升工程，新造林2.5万亩。开工建设中湖省级湿地公园及烈山、卧牛山生态恢复工程。
　　****促进节约和高效利用资源。****大力推行清洁生产，实施资源再生利用示范工程。支持建筑产业现代化发展，推广绿色建筑、建材，倡导合理、绿色消费。出台城市公共交通优先发展实施意见，鼓励绿色出行。全面推广应用节能新产品、新技术。实行水资源论证和用水定额管理制度，建设节水型城市。严守耕地保护红线，严格土地用途管制。
　　****（八）更加注重保障和改善民生，着力增强群众获得感****
　　****全力办好民生实事。****继续实施34项民生工程，新增农村道路畅通工程、重度残疾人护理补贴等项目，提高新农合、城镇居民医保、基本公共卫生服务、政策性农保、特教生均公用经费和校舍维修改造等5项补助标准。抓好高校毕业生、下岗失业人员、残疾人、退役士兵、失地人员等重点群体就业，为农民工返乡创业、就业培训、权益保障提供优质服务。
　　****坚决打赢棚户区改造攻坚战。****以改善市民居住条件为目的，抓住国家棚户区改造政策新机遇，积极争取利用国开行、农发行长期低息贷款，以老城区为重点，科学界定拆迁范围和地块，实行民主和谐拆迁和房票购房安置，倒排时间节点，严格调度考核，强力推动一县三区完成棚户区改造年度任务。新增各类保障性安居工程13142套。继续实施农村危房改造。
　　****完善社会保障和救助体系。****全面实施全民参保登记计划，建立完善社会保险数据库。按照政策及时调整各项社会保险待遇水平。加快城镇职工医保、城镇居民医保和新农合统筹发展，建立城乡统一的医疗保险制度。依法推进社会保险扩面征缴，扩大工伤保险覆盖范围。健全社会救助体制机制，鼓励、引导和动员慈善组织、志愿者、企业等各方力量通过多种方式参与社会救助。开展养老服务业综合改革试点，健全以五保供养为主的农村养老服务网络。
　　****提质发展教育事业。****完成学前教育第二期三年行动计划，全面提升学前教育普及水平。推进义务教育优质均衡发展，缩小城乡校际办学水平差距。支持和规范民办教育发展，做好新建小区配建义务教育学校工作。建立教师无校籍管理制度，扩大优质教育资源覆盖面，促进教育公平。围绕产业转型升级，整合职业教育资源，推动产教融合、校企合作。
　　****统筹发展社会事业。****全面推进市级公立医院综合改革。加强医疗联合体建设，推动优质医疗卫生资源合理配置。实施好全面两孩政策。落实全民健康行动计划，全力争创省级卫生城市。办好市第十届运动会。加快发展妇女儿童事业，健全农村留守儿童、妇女、老年人关爱帮扶体系。大力发展社会福利和慈善公益事业。做好第三次全国农业普查工作。加强气象、防空防震、防灾减灾工作。
　　****扎实推进精准扶贫。****实行“一村一策、一户一策”精准扶贫，确保全年减少贫困人口2万人以上。动员社会力量广泛参与扶贫事业，落实单位和干部定点帮扶机制，强化投入保障，提高扶贫成效。
　　****（九）深化社会治理创新，切实维护社会安全稳定****
　　****推进多层次多领域依法治理。****深化法治淮北建设，不断提升执法司法公信力。谋划“七五”普法规划，加快法治文化建设，不断提升法治宣传教育实效。深入开展依法治校、诚信守法示范企业、民主法治示范村（社区）、依法行政示范单位等法治创建活动，提高社会管理法治化水平。完善民族宗教事务治理体系。
　　****加强基层基础工作。****以争创全国社会治安综合治理优秀城市为目标，深入推进平安淮北建设，加快完善立体化数字化社会治安防控体系，依法严厉打击各类违法犯罪活动。持续推进基层综治维稳信访工作中心（站）规范化、社会服务管理信息化建设。落实重大决策社会稳定风险评估制度，继续推行重大不稳定问题清单制度，畅通信访渠道，坚持依法治访，最大限度减少矛盾的产生。
　　****强化安全生产和食品药品安全监管。****深入开展安全生产“铸安”行动和“六打六治”打非治违专项行动，推进事故隐患排查治理全覆盖，坚决遏制重特大安全事故。立足让市民吃得更放心、用药更安全，以争创省级食品药品安全城市为抓手，大力实施食品安全战略，强化依法监管、全程监管和社会共治。
　　****支持驻淮人民解放军、武警部队现代化建设。****推动国防动员和后备力量建设。深入开展“双拥在基层”和“四方共建”活动，全力争创全国双拥模范城。
　　****（十）注重改进和创新政府工作，不断提升服务水平****
　　****全面推进依法行政。****进一步规范政府系统重大事项决策行为，确保决策科学、程序正当、过程公开、责任明确。深化行政执法体制改革，推进重点领域综合执法，建立健全自由裁量权基准制度，严格规范公正文明执法。自觉接受人大及其常委会法律监督、工作监督和政协民主监督，认真办理人大代表议案、建议和政协委员提案。广泛听取社会各界意见，主动接受社会监督和舆论监督。
　　****不断创新政府管理。****健全政府购买公共服务机制，引导社会资本参与公益性事业投资运营。加快新型智库建设，健全决策咨询制度。全面推行政务公开，加强政务平台和政府网站信息化建设，扩大重点领域信息公开范围，及时回应社会关切。简化优化审批流程，推行网上办事，为群众办事创业提供更好的服务环境。
　　****持续提升行政效能。****始终把推动改革发展稳定作为最大的责任，夙夜为公、勤勉工作，勇于担当、敢于突破。深化精细作风建设，坚决整治“庸懒散拖”，坚决纠正不作为、慢作为、乱作为，推动政府工作提速提效。
　　****切实加强廉政建设。****认真落实中央八项规定精神，厉行勤俭节约，严防“四风”反弹。强化行政监察和审计监督，加大对财政资金分配使用、政府性投资、公共资源交易等重点领域监管力度。严格执行廉洁从政各项规定，认真履行“一岗双责”，驰而不息正风肃纪。
　　让我们在中共淮北市委的坚强领导下，齐心协力，开拓奋进，扎实工作，为建设精致淮北、夺取全面建成小康社会伟大胜利而努力奋斗！
